# Synthesis and Evaluation of Properties of an Additive Based on Bismuth Titanates for Cement Systems

**DOI:** 10.3390/ma16186262

**Published:** 2023-09-18

**Authors:** Svetlana V. Samchenko, Irina V. Kozlova, Andrey V. Korshunov, Olga V. Zemskova, Marina O. Dudareva

**Affiliations:** Department of Building Materials, Moscow State University of Civil Engineering, 26, Yaroslavskoye Shosse, 129337 Moscow, Russia; samchenkosv@mgsu.ru (S.V.S.); kozlovaiv@mgsu.ru (I.V.K.); korshunovav@mgsu.ru (A.V.K.); zemskovaov@mgsu.ru (O.V.Z.)

**Keywords:** cement composition, bismuth titanate, TiO_2_-Bi_2_O_3_ system, photocatalytic concrete, citrate synthesis, solid-state technology, photocatalytic activity, strength, nanotechnology

## Abstract

The development of modern building materials science involves the process of designing innovative materials that exhibit unique characteristics, such as energy efficiency, environmental friendliness, self-healing ability, and photocatalytic properties. This can be achieved by modifying cement with nano- and fine-dispersed additives that can give the material new properties. Such additives include a number of compounds based on the TiO_2_-Bi_2_O_3_ system. These compounds have photocatalytic activity in the near-UV and visible range of the spectrum, which can serve to create photocatalytic concretes. Here, the purpose of this scientific study was to synthesize compounds based on the TiO_2_-Bi_2_O_3_ system using two methods in order to identify the most optimal variant for creating a composite material and determine its properties. Within the framework of this article, two methods of obtaining a photocatalytically active additive based on the TiO_2_-Bi_2_O_3_ system are considered: the solid-state and citrate-based methods. The photocatalytic, mechanical and structural properties of composites containing the synthesized additive are investigated. In this study, it was found that for the creation of photocatalytic concretes, it is advisable to use cement compositions with a bismuth titanate content of 3–10 wt.%. of the cement content, regardless of the method of obtaining the additive. However, the most optimal composition is one containing 5 wt.% of the synthesized additive. It is noted that compositions containing 5% by weight of bismuth titanate demonstrate photocatalytic activity and also show an increase in strength on the first day of hardening by 10% for the solid-state method and 16% for the citrate method.

## 1. Introduction

The process of intensive development of the construction industry involves the creation of innovative materials that have a set of unique characteristics that exceed the properties of traditional building materials based on wood, polymers and inorganic binders. In addition, it involves expanding their scope of application: for instance, the use of aerogels as heat and sound insulation material [1,2], “smart” materials based on concrete and asphalt concrete with the self-healing ability [3,4], superhydrophobic coatings [5], energy-efficient glass [6], and conductive concrete [7]. Innovative functional materials must meet the maximum indicators of durability, energy efficiency, frost resistance, fire safety and decorativeness, showing a high degree of corrosion and biodeterioration resistance. All these qualities can be achieved in several ways, for example, by creating a multicomponent composite material, or by modifying a traditional material with various kinds of additives of organic, mineral, natural or industrial origin. In recent years, special attention has been paid to the modification of building materials with nanoobjects and nanostructures. These include nanoparticles of metals, oxides, carbon fibers and clusters, various ultra- and fine-dispersed additives, as well as surface nanomodification [8].

One of the most popular, versatile and multifunctional building materials nowadays is concrete-/cement-based materials that demonstrate high-quality characteristics, such as strength, density, resistance to aggressive environmental influences, and durability. A wide range has been developed of modifying additives that can affect the characteristics of cement stone or provide it with new unique properties: for instance, accelerators and retarders of setting, plasticizing additives that contribute to the regulation of the workability of the concrete mixture, or hydrophobic and air-entraining, pozzolanic, biocidal additives.

One of the relatively new types of modified cement composite material is photocatalytic concrete. Titanium dioxide with photocatalytic properties is introduced into its composition. TiO_2_ can exist in three allotropic modifications: rutile, anatase and brookite. Anatase modification has the maximum photocatalytic activity. Nanoscale TiO_2_ with a rutile:anatase ratio of 30:70 is used as an industrial photocatalyst Degussa P25 [9,10].

The uniqueness of photocatalytic concrete results not only in the increased aesthetic qualities of concrete modified with titanium oxide but also in its ability to oxidize pollutants deposited on the concrete surface. These include volatile organic compounds (VOCs), nitrogen oxides NO_x_ and sulfur oxides SO_x_, spores of microscopic fungi and bacteria.

In addition, titanium oxide particles provide the concrete surface with increased hydrophilic properties, so that the oxidation products of pollutants can be easily washed off with rainwater, enabling the surface to remain clean for longer. Thus, photocatalytic concrete can be used as a building material, or as a material for designing decorative panels or paving slabs, which, of course, is a promising direction for its study [11,12,13,14].

In recent years, the variety of heterogeneous photocatalysts has been significantly expanded: there are a number of works aimed at studying the photocatalytic activity of titanium oxide doped with metal and nonmetal atoms [15,16]: for instance, the photocatalytic properties of pure and doped zinc oxide [17,18], nanoscale cadmium sulfide CdS [19], graphite-like carbon nitride [20], cerium oxide CeO_2_ [21], CuO [22], BiOCl, SnO_2_, WO_3_ [23] as well as composite photocatalysts Fe_3_O_4_/TiO_2_ [24], CuBi_2_O_4_/polyaniline [25], BiFeO_3_-black TiO_2_ [26], layered CuO/ZnO [27], Dy_2_WO_6_-ZnO [28] and In_2_O_3_/SnO_2_ [29].

Previous authors [30,31,32,33,34,35] reported a number of compounds based on the TiO_2_-Bi_2_O_3_ system, which exhibit photocatalytic activity in the near-UV and visible range of the spectrum. Bismuth titanates of various compositions can be more effective as photocatalytic materials than that of TiO_2_ because of their narrower band gap energy: TiO_2_-based photocatalysts are active only under UV irradiation with λ > 382 nm, while for bismuth titanate-based compounds, the activity can be achieved in the visible spectra range: for Bi_2_Ti_4_O_11_, λ > 400 nm, for Bi_12_TiO_20_, λ > 427 nm, for Bi_2_Ti_2_O_7_, λ > 496 nm, and for Bi_4_Ti_3_O_12_, λ > 421 nm. This fact is due to the lower value of band gap energy (E_g_). For anatase TiO_2_, E_g_ is 3.2 eV, so its particles are active only under ultraviolet (UV) irradiation of λ < 387 nm, while for Bi_4_Ti_3_O_12_, E_g_ is 2.95 eV, for Bi_2_Ti_4_O_11_, E_g_ is 3.1 eV, for Bi_12_TiO_20_, E_g_ is 2.9 eV, and for Bi_2_Ti_2_O_7_, E_g_ is 2.5 eV.

Photocatalytic additives can be synthesized with various methods, such as solid-phase synthesis, sol-gel, the hydrothermal method, co-deposition method, or synthesis in the gas phase. After that, they can be used in the construction industry to obtain photocatalytically active materials and coatings.

Thus, based on the literature review and general trends in the construction materials science industry, the purpose of this scientific study was formulated, which was to synthesize compounds based on the TiO_2_-Bi_2_O_3_ system using two methods in order to identify the optimal variant for application in a composite material and determine its properties.

To achieve this goal, the following research objectives were formulated: to synthesize a photocatalytic additive for cement systems using solid-state and citrate-based methods, to investigate the photocatalytic activity of synthesized additives, and to study the mechanical and photocatalytic characteristics of cement stone modified with additive particles.

## 2. Materials and Methods

During the research in this work, at the first stage, an additive based on the TiO_2_-Bi_2_O_3_ system was synthesized using two different methods: solid-state synthesis from the corresponding oxides, and the citrate method, which allows us to obtain nanoscale particles. For the synthesis of the additive, oxides of titanium (IV) TiO_2_ and bismuth (III) Bi_2_O_3_ of analytical grade were used, which were ground in an agate mortar with the addition of isopropyl alcohol to improve homogenization, pressed into pellets and annealed in a muffle furnace at several stages with intermediate grindings in the temperature range of 650–800 °C. The total the annealing time was 24 h. To obtain the additive with the citrate method, titanium tetrachloride (TiCl_4_) = 1.72 g/mL), bismuth oxide of analytical grade, hydrochloric acid solution (1:1) and citric acid monohydrate were used. Titanium tetrachloride was added drop by drop to distilled water with constant stirring and cooling in an ice bath to form hydrolysis products. Bismuth oxide was dissolved in hydrochloric acid and a solution of citric acid was added; then, the resulting mixture was added to an aqueous solution of titanium tetrachloride. The resulting solution was evaporated in a water bath at 100 °C until a yellow gel-like solution was formed, which was decomposed in a muffle furnace at 400 °C (15 min) before the organic component burned out, and then annealed in a muffle furnace at a temperature of 700 °C until the final fine flake-like reaction product was formed.

**Figure 1:**
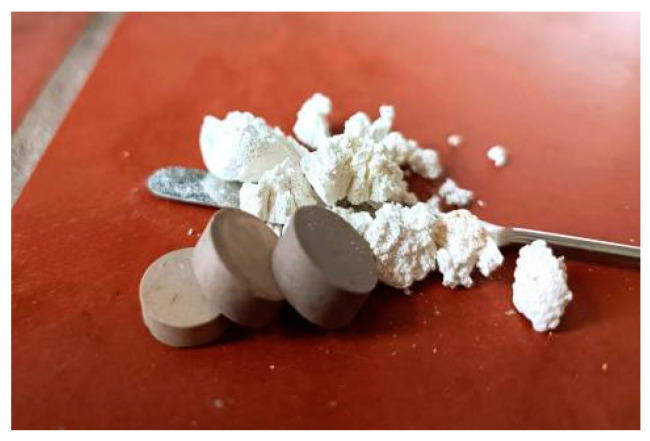
Photo of an additive obtained with solid-state method and citrate synthesis.

As a result of synthesis with solid-state reaction, dense pellets were obtained; as a result of citrate synthesis, a flake-like reaction product of light gray color was obtained (Figure 1).

**Figure 2:**
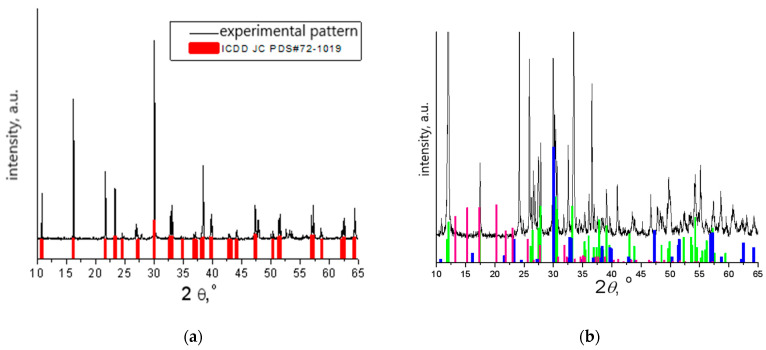
X-ray diffraction patterns of additives, obtained with (**a**) solid-state reaction and (**b**) citrate method: 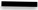 experimental data, 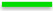 Bi_2_Ti_4_O_11_, 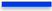 Bi_4_Ti_3_O_12_, 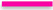 Bi_2_Ti_2_O_7_.

The phase composition of the bismuth titanate additive was studied using X–ray phase analysis, which was carried out using an X-ray diffractometer D8 ADVANCE (Bruker AXS, Karsruhe, Germany) CuKa radiation (graphite monochromator), λCuKa = 1.54056 Å. The X-ray diffraction patterns were processed using the Match! software version 3.15 Build 274 (Figure 2).

From the registered X-ray diffraction data patterns shown in Figure 2, it can be concluded that the main phase of the additive obtained with solid-state reaction corresponds to Bi_4_Ti_3_O_12_ (PDF#72-1019). The main compound in the additive sample, synthesized with the citrate method, corresponds to Bi_2_Ti_4_O_11_ (PDF#83-0673), but there is also a Bi_4_Ti_3_O_12_ (PDF#72-1019) phase with a perovskite structure and a metastable phase Bi_2_Ti_2_O_7_ (PDF#32-0118) with a pyrochlore structure.

**Figure 3:**
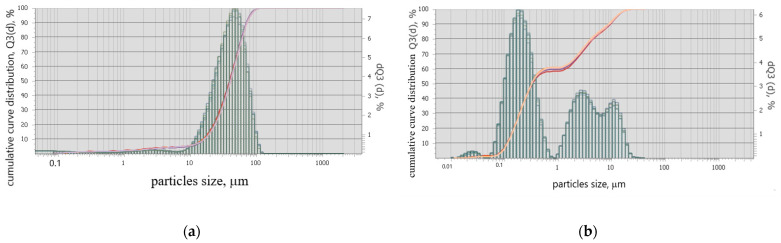
Particle size distributions of the additive, synthesized via (**a**) solid-state method and (**b**) citrate-based method.

A granulometric composition analysis of the additives synthesized with two methods was performed on a Analysette 22 NanoTec device (Fritsch, Germany). Figure 3 shows that the average particle size of the additive obtained via the solid-state method is 50 μm, while for the citrate-based method the average particle sizes are 0.33, 5 and 10 μm.

The photocatalytic properties of the additive were studied via decomposition of the model dye Rhodamine B: a sample of photocatalysts m = 50 mg was introduced into 25 mL of Rhodamine B solution with a concentration of 2 × 10^−5^ mol/L, and then subjected to ultrasonic processing on a UZDN-1 device at a frequency of 44 kHz for 15 min in a dark place to establish adsorption equilibrium. Then, the resulting suspension of photocatalysts with constant stirring using a magnetic stirrer was irradiated under a UV lamp (λ = 365 nm “Black light”, 26 W, 50 Hz) within 40 min. A sample of the solution was taken every 5 min, followed by separation of the photocatalyst particles on a laboratory centrifuge (10 min, 10,000 rpm) and determination of the optical density of solutions with a residual dye concentration on the photoelectric colorimeter KFK-2.

Optical absorption properties play an important role in photocatalytic activity determination. To determine the absorption of samples in the UV-Vis range of the spectrum, a water–glycerin suspension of additive particles was prepared, and the SF-2000 Spectrophotometer device (Spectr, Moscow, Russia) was used.

To study the photocatalytic activity of cement stone samples, as well as to study the properties of cement paste and physical and mechanical parameters of the obtained cement stone samples modified with a photocatalytic additive, white cement Cemix PRO WHITE 1-500 “Cemix Russia” (Lasselsberger group GmbH, Pöchlarn, Austria) was used (further denoted as WPC); its chemical and mineralogical compositions are presented in Table 1 and Table 2.

## 3. Results

**Figure 4:**
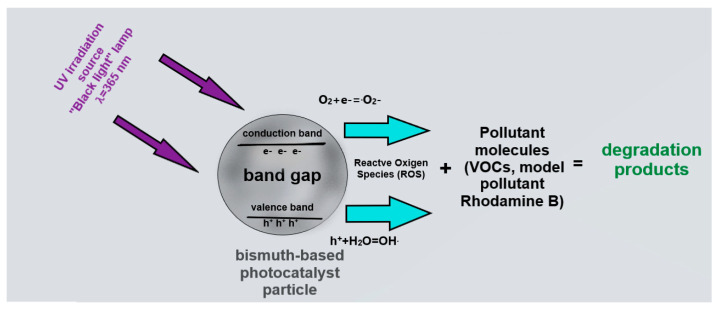
Schematic representation of the photocatalytic process.

The principle of photocatalysis is based on irradiation using light with energy sufficient to transfer an electron from the valence band to the conduction band. As a result of the course of secondary reactions, reactive oxygen species (ROS) capable of decomposing pollutants are formed on the surfaces of the particles (Figure 4).

**Figure 5:**
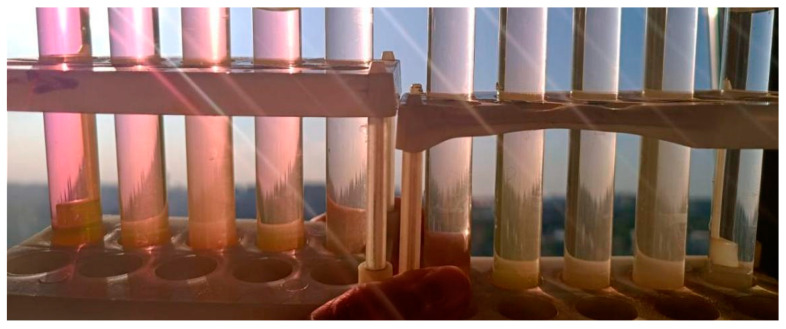
Visual reduction in the color intensity of the Rhodamine B solution after a photocatalytic reaction of the additive obtained via citrate method.

**Figure 6:**
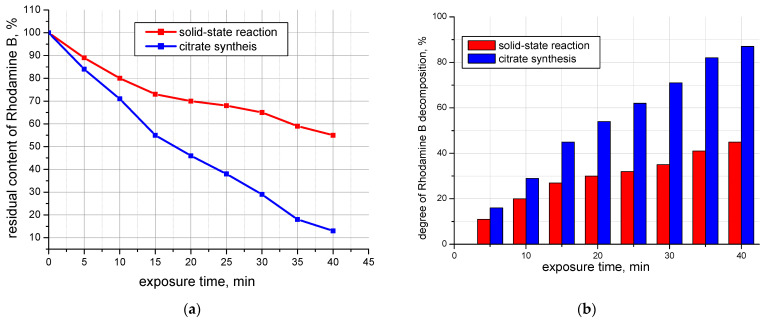
Residual content of Rhodamine B dye (**a**) and degree of Rhodamine B decomposition (**b**) after exposure to UV radiation.

To study the photocatalytic activity of the additive, an experiment was conducted on the color intensity loss of the organic dye Rhodamine B (Figure 5 and Figure 6), which was carried out as follows: 50 mg of the additive was introduced into a 25 mL solution of Rhodamine B with a concentration of Cm = 2∙10^−5^ mol/L. This was subjected to ultrasonic treatment on the UZDN-1 device at a frequency of 44 kHz for 15 min in the dark to establish adsorption equilibrium. Then, while stirring with a magnetic stirrer, the suspension was irradiated under a UV lamp (λ = 365 nm “Black light”, 26 W, 50 Hz) for 40 min, taking a sample every 5 min, with determination of the optical density of the solution on the photoelectrocolorimeter KFK-2 after separation of residue from filtrate on a laboratory centrifuge.

As can be seen from Figure 6, the photocatalytic activity of the additive synthesized with the citrate method is higher than that obtained with the solid-state method. Probably, this fact is related to the particle size of the photocatalysts: the more highly developed the surface of the photocatalyst, the more active centers can be involved in the process of adsorption of dye molecules and its photocatalytic oxidation.

**Figure 7:**
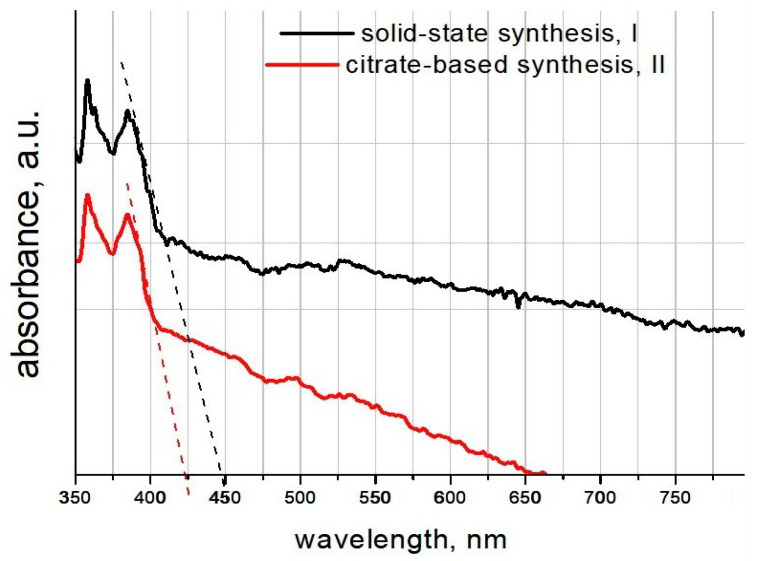
UV-Vis absorption spectra of the synthesized additives.

Figure 7 displays the UV-Vis absorption spectra of the additive samples, obtained via solid-state (black line, I) and citrate-based (red line, II) methods at the wavelength in the range of 350–800 nm.

The spectra were obtained by subtracting the spectrum of the comparison solution (water + glycerin); that is, the UV part of the water absorption spectrum was removed.

The band gap absorption edges of I and II additives were determined to be 451 nm and 425 nm, respectively, which demonstrated visible light absorption. The band gap (E_g_) was calculated from the following equation:

$$Eg_{\;}=\frac{hc}{\lambda }\;,$$

where E_g_ is the optical band gap, *h* is Planck’s constant, λ is the wavelength corresponding to the onset of absorbance, and *c* is the velocity of light. Thus, the calculated E_g_ values of I and II additive samples were 2.75 and 2.91 eV, respectively. These results indicated that the additives demonstrated photocatalytic ability in the visible spectra region.

After establishing the photocatalytic activity of the synthesized additives, cement stone samples containing these additives were obtained. The modified samples were square-shaped plates with a side length of 5 cm. Bismuth titanate additives were introduced into the cement by dry mixing of components in the amounts of 0.3; 1.0; 1.7; 3.0; 5.0; and 10.0% additive by weight of the binder. The resulting compositions were mixed with tap water and left to harden under standard conditions.

**Figure 8:**
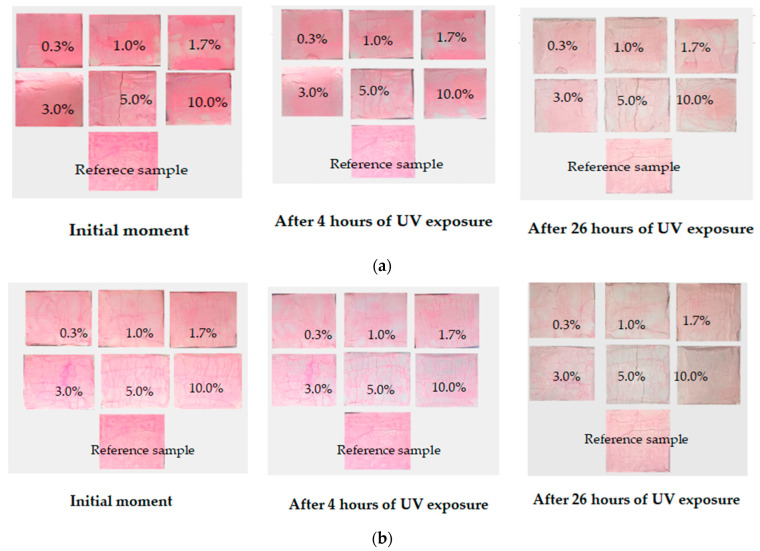
Change in the color of Rhodamine B after UV exposure on the surface of samples modified with an additive obtained with (**a**) solid-state or (**b**) citrate method.

**Figure 9:**
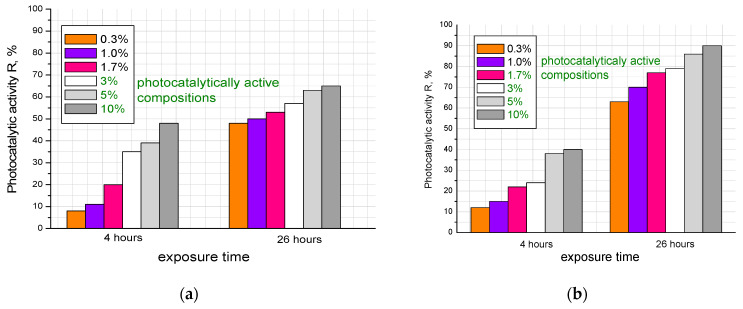
Photocatalytic activity of cement stone samples modified with an additive obtained with (**a**) solid-state or (**b**) citrate method.

The photocatalytic activity of the obtained cement stone samples was evaluated in accordance with the Italian standard UNI 11259-2016 [36], which consists of fixing the discoloration (mineralization) of the organic pigment Rhodamine B applied to the surface of concrete modified with a photocatalyst when exposed to UV radiation after 4 and 26 h (Figure 8 and Figure 9). The experiment was carried out as follows: the obtained cement stone samples were placed in a special container; then, a solution of the organic dye Rhodamine B with a concentration of 4 × 10^−4^ mol/L in an amount of 1 mL was applied to the surface of the samples and exposed to UV radiation. The degree of photocatalytic activity of cement composites was assessed by photographing the surface of the samples at the initial time, and then after 4 and 26 h of exposure under UV light. The processing of the data obtained from the photographs was carried out using ImageJ software version 1.54d, which was used to convert the color parameter R of the RGB space into the LAB color model, where L is the brightness of the object; a is the axis along which the gradations from red to green are postponed; and b is the axis with gradations from yellow to blue. The photocatalytic activity of cement stone samples was evaluated by changing parameter a and calculated using the following formulas:

(1) $$R4=\frac{a0-a4}{a0}\times 100\%\;\;\;\;\;\;\;\;\;\;\;\;\;\;\;\;\;\;R26=\frac{a0-a26}{a0}\times 100\%\;$$

where *a*0 is the value of the color coordinate at the initial time; *a*4 is the value of the color coordinate after 4 h of UV radiation; and *a*26 is the value of the color coordinate after 26 h of UV radiation. The R values should be more than 20% after 4 h and more than 50% after 26 h of exposure to UV radiation, according to the UNI 11259 standard, which determines whether concrete exhibits photocatalytic activity.

Thus, from the data presented in Figure 8 and Figure 9, it can be concluded that samples modified with the additive obtained with the citrate method had greater photocatalytic activity with respect to the mineralization of the organic pigment Rhodamine B. This applied to photocatalytically active samples with additive concentrations of 1.7; 3.0; 5.0; and 10 wt.%. The additive-free sample and composites with 0.3 and 1.0 wt.% additives did not show activity. The observed slight loss of surface color intensity was most likely due to drying and exposure to UV radiation. Samples modified with the additive synthesized with the solid-state method were active with a larger amount of the additive, starting from 3 wt.% (3.0, 5.0 and 10%). This can probably be explained by the size of the additive particles and the difference in the specific surface area of the samples: the particles obtained with the solid-state method were larger, fewer of them appeared on the surfaces of the samples, and the catalytic activity was lower.

The effect of the synthesized additive on the properties of the cement paste was also investigated (Table 3), and an acceleration of the setting time and an increase in the normal consistency of the cement paste were revealed when additives were introduced in the amounts of 1.0; 3.0; 5.0; and 10.0% by weight. 

This can be explained by the fact that fine particles of the additive, being an inert substance that does not exhibit its own hydraulic activity, serve as a substrate for the origin and growth of compounds forming during the hydrolysis and hydration reactions of clinker cement minerals.

**Figure 10:**
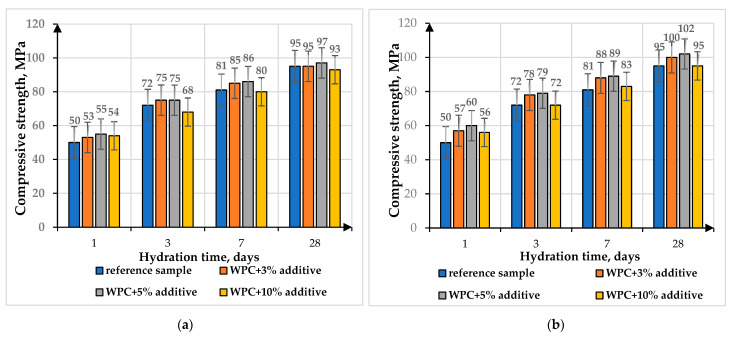
Compressive strength of samples with synthesized additive: (**a**) solid-state method; (**b**) citrate method.

The mechanical properties of the modified composites were studied on cube samples of 2.0 × 2.0 × 2.0 cm using the Controls laboratory hydraulic press, thereby determining the compressive strength at the ages of 1, 3, 7 and 28 days (Figure 10). To obtain modified cement stone samples in accordance with Table 3, the average water–cement ratio (W/C = 0.36) was selected.

From the data presented in Figure 10, it follows that when the additive is introduced in quantities of 3–5 wt.%, an increase in the strength of the samples is observed: in the initial periods of hardening by 4–8% (for solid-state reaction additive) and by 9–17% (for citrate method additive), the strength of the samples is at the level of the control sample at 28 days of age. With the introduction of 10 wt.% of additive, a slight decrease in strength by 4–6% for the solid-state reaction additive and by 1–3% for the citrate method additive is observed at 28 days of age. The obtained results of samples with the addition of bismuth titanates were comparable with the reference sample, which indicated that the addition of bismuth titanates up to 10 wt.% did not worsen the strength characteristics of the samples significantly.

**Figure 11:**
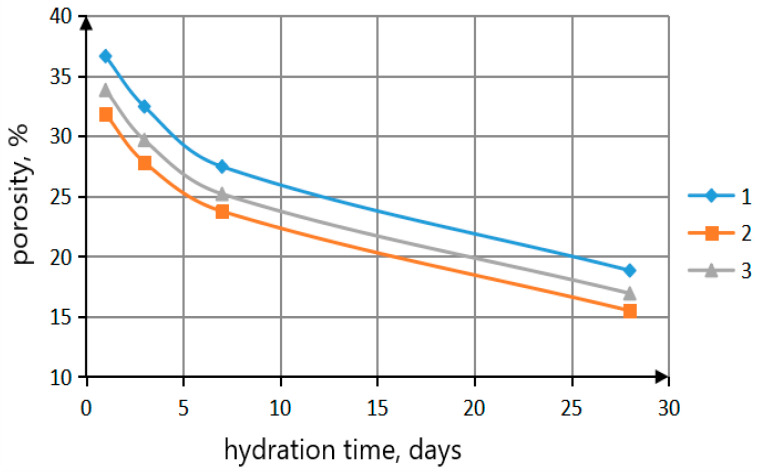
Dependence of porosity of cement stone on hydration time: (1)—control sample; (2)—cement stone containing bismuth titanates in an amount of 5.0 wt.% synthesized via citrate-based method; (3)—cement stone containing 5.0 wt.% bismuth titanates synthesized via solid-state method.

The determination of porosity of cement samples was conducted using the pycnometer method (Figure 11).

During the study of the cement stone structure, it was found that porosity was lower in samples containing bismuth titanate particles, regardless of the synthesis method, compared with the control sample: on average by 10% on the first day and by 14% at 28 days of age. Porosity values of cement stone samples modified with the additive synthesized via the citrate method were lower than for the samples with the additive obtained via the solid-state reaction. This is due to the fact that the particle size of the additive synthesized with the citrate-based method is much smaller than the particle size of the additive obtained with the solid-state method. The smaller particle size of the additive contributes to the more active process of clinker mineral hydration. The particles of the additive serve as centers of crystallization for the formation and growth of crystallohydrates, which makes the structure of the cement stone denser. 

The obtained strength and structural characteristics in combination with photocatalytic properties confirm the possibility of creating photocatalytic concrete.

## 4. Discussion

Within the framework of this research the authors evaluated the photocatalytic activity of additives for cement composites based on TiO_2_-Bi_2_O_3_ system obtained via solid-state and citrate-based methods. It can be concluded that the additives exhibit photocatalytic activity with respect to the decomposition of the organic pigment Rhodamine B when irradiated with near-UV light. Thus, as a result of the conducted studies, the photocatalytic activity of an additive based on the TiO_2_-Bi_2_O_3_ system under the influence of UV radiation is known. Furthermore, the possibility of obtaining cement composites modified with particles of an additive obtained using the solid-state or citrate method and their photocatalytic activity with respect to the mineralization of the organic pigment Rhodamine B under UV light were established. The evaluation was carried out in accordance with the Italian standard UNI 11259-2016. Compositions based on white cement exhibiting photocatalytic activity were determined: the concentrations of the additives obtained with the solid-state method were 3.0; 5.0; and 10 wt.%, while with the citrate method, they were 1.7; 3.0; 5.0; and 10 wt.%. Apparently, this fact is explained by the particle size: during solid-state synthesis, larger particles are formed than in the citrate method; thus, a greater number of active particles appears on the surface of the modified cement stone. In addition, an assessment of the cement paste properties and mechanical characteristics of the modified cement stone in comparison with the reference sample was carried out. As a result, it was revealed that the maximum increase in strength was observed for samples containing 5 wt.% of additives, especially on the first day. For the additive synthesized with the solid-state method, the compressive strength increased by 10%, compared to 16% for samples modified with an additive obtained using the citrate method. At 28 days of age, the compressive strengths of the modified and reference samples were almost the same. Despite the fact that the introduction of 10 wt.% of additive obtained via either the solid-state method or the citrate method slightly reduced the compressive strength of samples at 28 days of age by 3–5%, in general, it can be noted that the introduction of bismuth titanates in an amount of 3–10 wt.% into the cement system is comparable to the reference sample. In combination with photocatalytic properties proven in accordance with European standard UNI 11259-2016, this allows us to consider the resulting compositions as applicable for the production of photocatalytic concrete and cement-based materials.

## 5. Conclusions

In the course of the research, an additive based on the TiO_2_-Bi_2_O_3_ system was synthesized, which has photocatalytic properties in the near-UV spectral range. The synthesis of the additive was carried out using two methods: solid-state and citrate synthesis. In accordance with the European standard UNI 11259-2016, the obtained additive compositions exhibit photocatalytic properties. However, the addition of bismuth titanates obtained using the citrate method gives higher photocatalytic activity. Nevertheless, we believe that both methods considered can be used to obtain photocatalytic composites.

Within the framework of the conducted research, the properties of cement paste and mechanical properties of cement stone samples were studied. It was found that the introduction of bismuth titanate additives, obtained by either the solid-state method or the citrate method, accelerated the initial and final setting periods of the cement paste, which correlates with an increase in compressive strength in the first day of the samples. There is also an increase in the water demand of cement paste modified with a synthesized additive. This is due to the fact that the additive is finely dispersed and the more it is contained in the cement system, the more water is required for the cement system itself.

When considering the physical and mechanical characteristics of cement stone containing an additive of bismuth titanates, an increase in compressive strength was noted on first day for all samples, regardless of the synthesis method. With further hardening time, the best compressive strength results were shown by samples containing 5 wt.% of bismuth titanates. Samples with 10 wt.% of the additive showed the lowest results in compressive strength, but they were insignificant (strength decreased at 28 days of age by 3–5% compared to the reference sample), which allows us to consider this composition in further studies. It is also noted that the addition of bismuth titanates obtained with the citrate method led to higher photocatalytic, physical and mechanical properties, which is associated with the formation of more finely dispersed particles. Despite the fact that there was some superiority of properties when obtaining the additive of bismuth titanates using the citrate method over the solid-state method, we believe that it is possible to use either of the two considered additive production methods to obtain photocatalytic concretes. To obtain photocatalytic concretes, we recommend using compositions containing an additive of bismuth titanates in an amount of 3–10 wt.%. When considering the price–quality issue, the composition containing 5 wt.% of additives is optimal because it combines good photocatalytic parameters with enhanced strength characteristics; therefore, it can be used for designing photocatalytic cement compositions. Future studies. In future studies, the authors plan to investigate the photocatalytic performance of the cement stone modified with synthesized additive in the natural environment, as well as the reusability of the systems.

## Figures and Tables

**Table 1:** Chemical composition of clinker “Cemix PRO WHITE 1-500”.

Clinker Components	Calcination Loss, %	CaO	SiO_2_	Al_2_O_3_	Fe_2_O_3_	MgO	SO_3_	R_2_O
%	1.0	65.0	21.0	5.5	0.5	1.0	3.3	0.1

**Table 2:** Mineralogical composition of clinker “Cemix PRO WHITE 1-500”.

Mineral Content, %			
C_3_S	C_2_S	C_3_A	C_4_AF
66.0	8.5	13.5	1.5

**Table 3:** Normal consistency and setting time of cement paste modified with the additive.

Additive Content, wt.%.	Normal Consistency, % Solid-State/Citrate	Setting Time, h min Solid-State/Citrate		Acceleration (+)/Slowdown (−) of Setting Time, min Solid-State/Citrate	
		Initial	Final	Initial	Final
-	34.3	2-00	3-30	-	-
0.3	34.3/34.3	2-00/2-00	3-30/3-30	-/-	-/-
1.0	34.5/34.6	1-55/1-55	3-25/3-25	+5/+5	+5/+5
1.7	34.6/35.0	1-55/1-50	3-25/3-25	+5/+10	+5/+5
3.0	34.8/35.3	1-50/1-50	3-25/3-20	+10/+10	+5/+10
5.0	35.0/36.0	1-45/1-40	3-15/3-15	+15/+20	+15/+15
10.0	37.5/38.0	1-35/1-35	3-05/3-05	+25/+25	+25/+25

## Data Availability

The data presented in this study are available on request from the corresponding author.
